# Effects of balance training with electrical muscle stimulation on ankle joint muscle on sensory reweighting and motor strategy

**DOI:** 10.1186/s12984-026-02114-3

**Published:** 2026-08-06

**Authors:** Masato Shindo, Ryosuke Aoki, Naoya Hasegawa

**Affiliations:** 1 NTT Human Informatics Laboratories, NTT, Inc., 1-1 Hikari-no-oka, Yokosuka, Kanagawa 239-0847 Japan; 2 Graduate School of Health Sciences, Hokkaido University, N12-W5, Kitaku, Sapporo, Hokkaido 060-0812 Japan; 3 Department of Rehabilitation Science, Faculty of Health Sciences, Hokkaido University, N12-W5, Kitaku, Sapporo, Hokkaido 060-0812 Japan

**Keywords:** Augmented sensory feedback, Balance training, Electrical muscle stimulation, Postural control, Sensory reweighting

## Abstract

****Purpose**:**

Augmented sensory feedback is expected to improve postural balance through modulation of sensory reweighting dynamics. Electrical muscle stimulation (EMS) directly assists joint movement via involuntary contractions, potentially influencing both sensory and motor systems in postural control. However, it remains unclear whether EMS-based balance training specifically facilitates adaptive changes in sensory weighting, and if so, how it modifies postural control strategies. In this study, we therefore examined the effects of balance-board training with EMS applied to the ankle muscles on sensory reweighting dynamics and postural strategies in healthy adults.

****Methods**:**

Forty healthy participants performed a static standing (ST) task under four surface–vision conditions and a balance-board (BB) task requiring horizontal stabilization of the board, before and after a five-minute balance-board training session. During training, the EMS group (n = 20) received stimulation of the ankle dorsiflexor or plantarflexor muscles depending on board tilt, while the control group (n = 20) practiced without EMS. Postural outcomes were assessed using center of pressure (COP; ST task only), body segment kinematics using motion capture, and electromyography of the ankle and hip muscles. Pre–post and between-group comparisons were conducted

****Results**:**

After training, the EMS group showed increased power spectral density of the low-frequency band in the ST task, indicating increased reliance on vision. They also exhibited greater use of hip movements with shoulder stabilization, reduced coupling between ankle joint angle and COP displacements, and decreased hip muscle co-contraction compared to the control group, suggesting that EMS induced a shift toward a more coordinated hip-centered postural strategy.

****Conclusions**:**

EMS-based balance training was associated with the induction of visual reweighting dynamics and adaptive postural strategies involving multiple joints. Specifically, EMS-induced involuntary ankle assistance elicited hip-centered adjustments in postural control, contributing to upper-trunk stabilization and increased reliance on visual inputs for postural balance regulation. These findings provide a foundational knowledge for training methods that support the learning of postural control using visual information. For establishing novel balance training tailored to individual sensory characteristics, future research should explore other augmented feedback techniques that can induce diverse forms of sensory reweighting.

## Background

Improving human postural balance is essential for preventing falls. Human postural control is performed by the integration of sensory information, primarily visual, somatosensory, and vestibular inputs, which are processed by the central nervous system to regulate muscle contractions and joint torques [1–3]. In this sensory integration process, a mechanism known as sensory reweighting adjusts the relative reliance on each sensory modality according to the surrounding environment and the internal state of the body. Inappropriate sensory dependence can disturb postural stability and increase the risk of falling [4–6]. Previous studies have shown that the dynamics of sensory reweighting vary across individuals, even within the same population (e.g., healthy, elderly, or stroke patients) [7–10]. Therefore, in postural balance training, it is crucial to adopt approaches that enhance stability by taking into account individual variability in sensory reweighting dynamics.

Augmented sensory feedback has been shown to improve postural balance [11]. Augmented feedback systems for postural control aim to provide additional sensory information by externally presenting body sway data (e.g., real-time center of pressure (COP) displacement or kinematic signals) through sensory modalities. Various types of feedback, including visual [12, 13], auditory [13–15], and tactile (vibrotactile [16] and electrotactile [17]) modalities, have been proposed to enhance postural control not only in healthy individuals but also in patients with neurological disorders. Recent studies have quantitatively demonstrated that such augmented sensory feedback also influences the dynamics of sensory reweighting. For example, Bao et al. suggested that vibratory stimulation to the lower trunk at the L4 / L5 level can increase vestibular contribution [16], and our own previous study showed that the induced reweighting dynamics vary depending on the modality used in the augmented feedback system [18, 19]. On the basis of these findings, we feel that specifying the type of sensory feedback could lead to a unique training program capable of inducing targeted reweighting dynamics. To validate this approach, it is necessary to investigate both the reweighting tendency induced by each feedback method and the associated learning effects on postural control.

Among various feedback techniques, electrical muscle stimulation (EMS) has received the most focus because, in addition to providing sensory cues, it has the distinctive feature of directly assisting joint movement through involuntary muscle contractions [20–26]. Systems that apply EMS to the ankle dorsiflexor or plantarflexor muscles during standing have been reported to improve postural balance in a wide range of individuals, from healthy participants [26] to people with physical disabilities [25], suggesting its potential as a training tool. We previously proposed a method in which EMS is delivered to the dorsiflexor or plantarflexor muscles according to the tilt of a balance board while standing on the board [18]. Our findings suggested that individuals who showed greater improvements in balance during stimulation increased their reliance on vision. However, it remains unclear whether EMS-based training specifically facilitates adaptive changes in sensory weighting across the three modalities, and if so, how it influences broader postural control strategies, including multi-joint coordination and muscle activity.

In evaluating the adaptive effects of balance training on sensorimotor strategies, it has become increasingly important not only to assess linear variability metrics, such as sway velocity and amplitude, but also to analyze dynamic aspects of posture through nonlinear variability metrics, including time–frequency analysis and sample entropy. Time–frequency analysis of the COP while standing provides valuable insight into sensory contributions, with power spectral density (PSD) reflecting the weighting of sensory inputs [27–31]. In addition, sample entropy is utilized to estimate the degree of “complexity” in physiological systems, where higher entropy values indicate a greater degree of complex dynamics [32, 33]. Beyond its application to physiological deficits and muscle fatigue [34, 35], recent studies have also used sample entropy to interpret the adaptive effects of balance training [34, 36, 37]. Therefore, to comprehensively understand both sensory reweighting and postural strategy processes in postural control, it is essential to incorporate these metrics.

In the current study, we investigate the adaptive effects of standing balance training with EMS applied to the ankle muscles in healthy participants, in order to examine the fundamental effects of EMS on sensory reweighting dynamics and postural strategy under controlled conditions. Based on our previous findings [18, 19], we speculate that involuntary assistance of ankle joint movements would require adaptive responses from the knee, hip, and trunk, leading participants to rely more on visual information than on ankle proprioception. Therefore, we hypothesize that EMS-based balance training would increase the visual contribution in postural control. Furthermore, we assume that this effect would be associated with the unique characteristics of EMS, which directly assists ankle joint motion through involuntary muscle contractions and thereby modify multi-joint coordination patterns during postural control.

## Methods

### Participants

Forty healthy adults without neurological or musculoskeletal disorders participated in this study and were randomly assigned to the EMS group (n = 20, 10 men and 10 women, age = 34.2 ± 9.3 years old) or the control group (n = 20, 10 men and 10 women, age = 32.8 ± 7.6 years old). Prior to the experiment, they were informed of the purpose of this study, the protocol, and potential risks, and signed a written consent form. The study was conducted in accordance with the Declaration of Helsinki (1964), and was approved by the ethics committee of the Cybernetics Laboratory of NTT Human Informatics Laboratories (approval number: 2023-08).

### Protocol

**Fig. 1:**
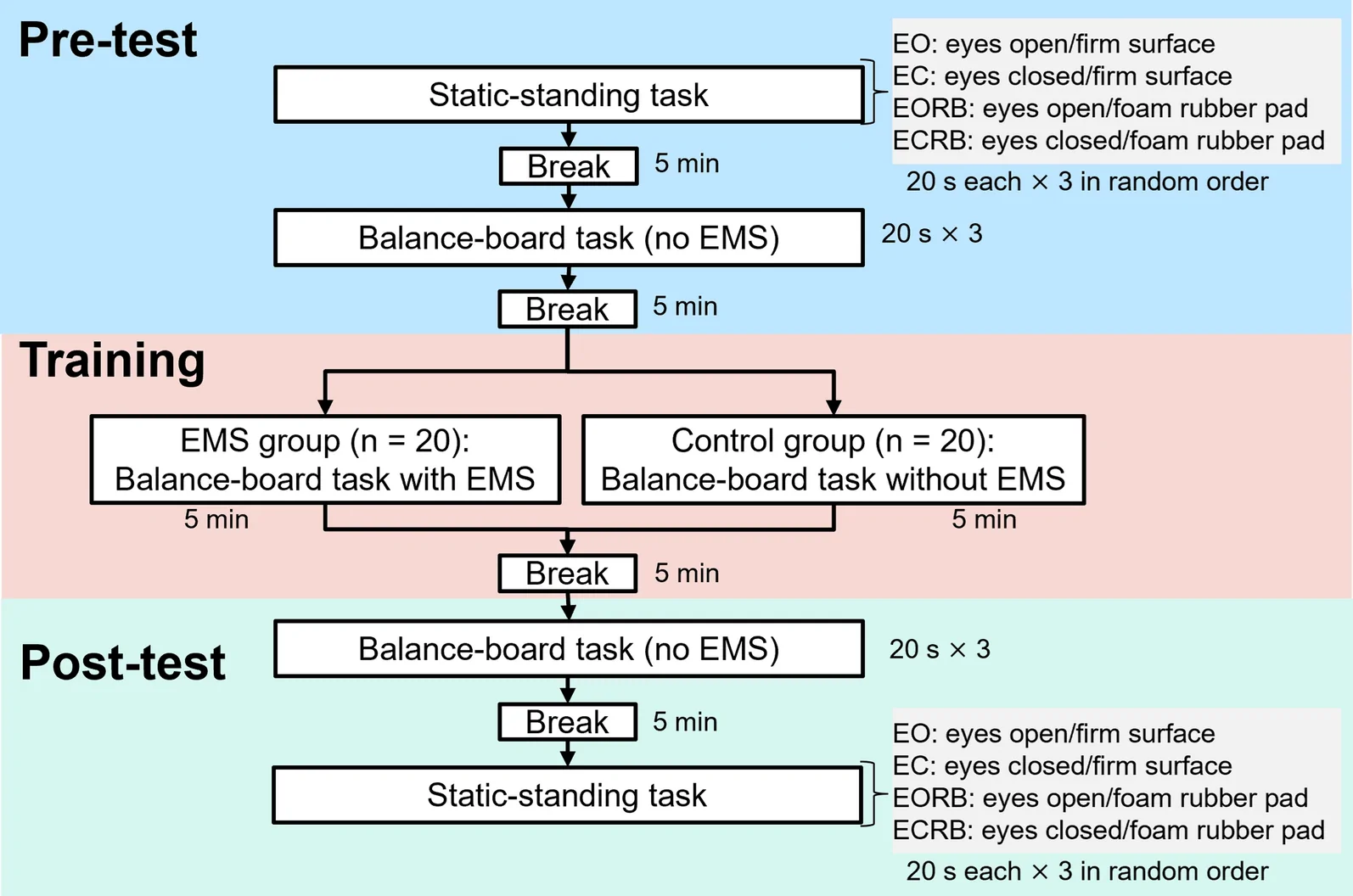
Experimental flow

As shown in Fig. 1, the experimental protocol comprised a pre-test, training, and post-test. The pre- and post-test included a static-standing task (ST task) and a balance-board task (BB task). In the pre-test, the ST task was performed first, and in the post-test, the BB task was conducted first, to examine immediate changes in sensory contributions assessed during the ST task following the BB task. In the training, participants practiced standing on the balance board for five minutes. The EMS group practiced while receiving EMS on the ankle muscles of both feet, whereas the control group practiced without EMS.

**Fig. 2:**
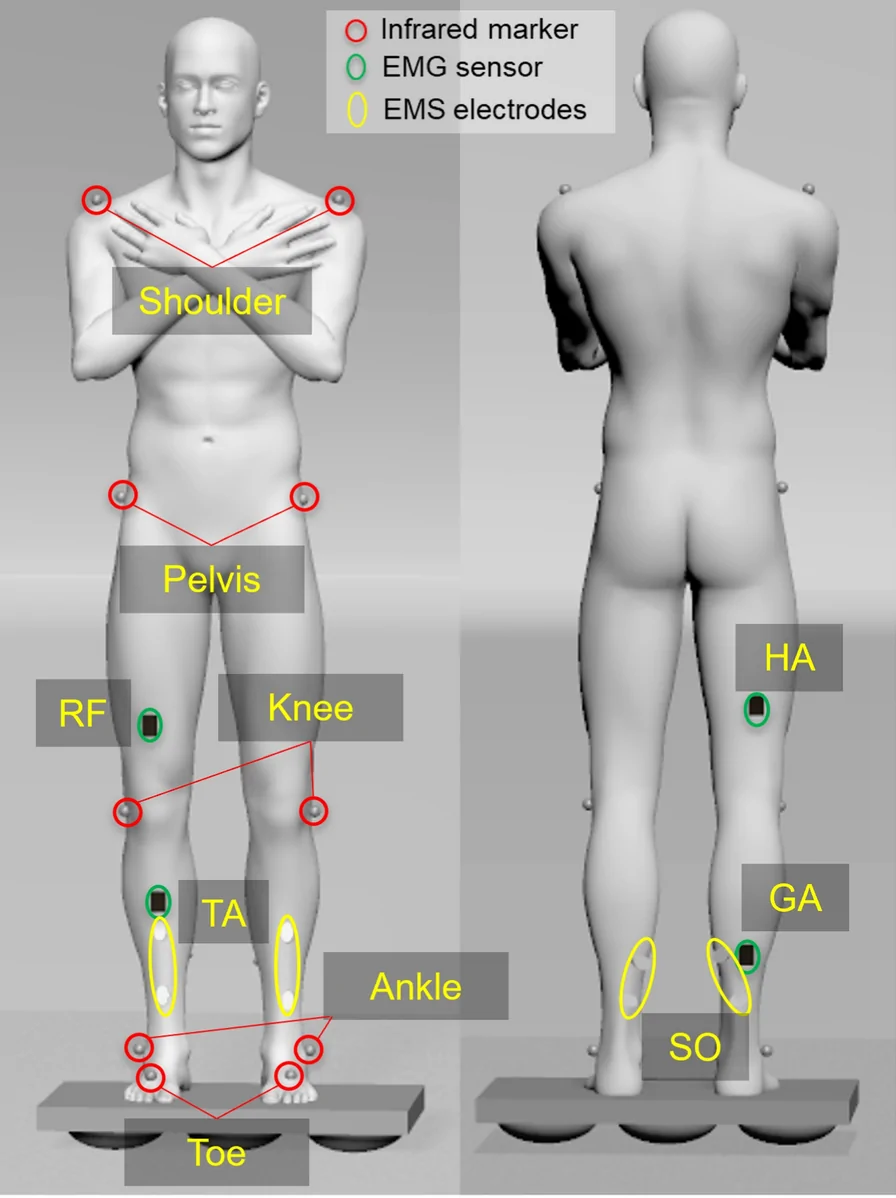
Positions of EMG sensors, EMS electrodes (only EMS group), and balance-board markers

Before the experiment, motion capture markers and electromyography (EMG) sensors were attached to the locations shown in Fig. 2. The motion capture markers were attached to ten anatomical locations on the participants’ bodies: the left and right shoulders (acromioclavicular ligaments), pelvis (iliac crests), knee joints, ankles (lateral malleolus), and the toes (second metatarsal bones). Additional markers were attached to the front, middle, and back ends of the balance board. EMG sensors (Delsys, Trigno Wireless System) were placed on four muscles of the participant’s right lower limb: the tibialis anterior (TA), gastrocnemius (GA), rectus femoris (RF), and hamstrings (HA).

#### ST task

The ST task adopted the protocol of the modified sensory organization test (mSOT), in which static standing was performed under the following four conditions with different surface and visual settings [6, 8, 38]. This task assessed the contribution of sensory inputs to postural balance and the ability to maintain static standing.

1. Eyes open on a firm surface (EO): All three sensory systems (visual, vestibular, and proprioceptive) contribute to balance.
2. Eyes closed on a firm surface (EC): Visual input is removed, requiring reliance on proprioceptive and vestibular information.
3. Eyes open on a foam rubber pad (EORP): Proprioceptive input is compromised due to the unstable surface, increasing reliance on visual and vestibular cues.
4. Eyes closed on a foam rubber pad (ECRP): Both visual and proprioceptive inputs are unreliable or absent, making the vestibular system the primary source of balance control.

Participants performed each condition barefoot, standing either directly on the force plate (BP400600, AMTI) for the EO and EC conditions or on a foam rubber pad (410 *× * 500 *× * 60 mm, blue foam – Airex®, AIREX [39]) placed on the plate for the EORP and ECRP conditions. They were instructed to stand with their heels together and their toes pointing outward at ${45}^{\circ }$, keeping their hands on their chest and maintaining a stable posture without movement. During the EO and EORP conditions, participants focused on a black area positioned at eye level approximately 2 m in front of them. Each trial lasted 20 s, and each condition was performed three times. The order of conditions was randomized across participants. If a participant lost balance, the trial was immediately stopped to prevent a fall and then repeated.

#### BB task

In the BB task, the participants stood upright in a double-leg stance on a balance board (300 *× * 500 *× * 30 mm, Dyjoc board, SAKAI Medical) and were asked to keep the board as horizontal as possible. Their feet were positioned parallel and approximately shoulder-width apart. Participants were instructed to cross their arms in front of their chest and look at a black dot on the front monitor while maintaining their stance. The board’s center was positioned at approximately 40% of the foot length from the heel line, following a previous study on COP during static standing on a stable surface [40]. This placement minimizes ankle joint moments when the board is level, meaning that maintaining a horizontal position relies on coordinated ankle muscle activity. The board was designed to tilt only in the anteroposterior direction due to three conical components attached to the bottom: two smaller components (80 mm in diameter, 60 mm in height) positioned on the left and right edges, and one larger component (160 mm in diameter, 60 mm in height) positioned at the center. Parallel bars (TB-534-02, Takada bed) were placed on both sides for safety. Each trial lasted 20 s, and each condition was performed three times. The data collection started after the participant released their hands from the parallel bars and assumed the measurement posture. Before the BB task in the pre-test, participants practiced the task for 30 s to ensure they could maintain balance and initially familiarize themselves with the task. If the participants could not maintain their posture, they grabbed the parallel bars to prevent falling, and the trial was immediately stopped and then repeated.

### EMS-based balance training

**Fig. 3:**
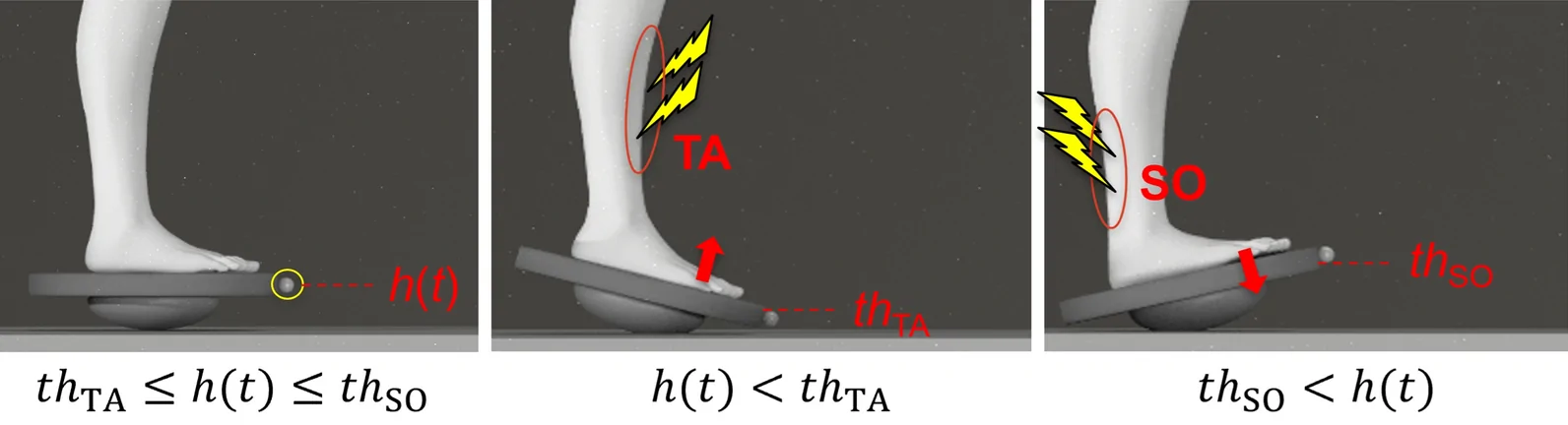
Schematic diagram of stimulation methods for participants in EMS group

In the training, participants in the EMS group practiced standing on the balance board while receiving EMS on the ankle muscles of both feet. The participants were equipped with four pairs of disposable electrodes (F-150 M, Nihon Kohden), one for the TA and one for the soleus (SO) of each leg (Fig. 2). The SO was selected instead of the GA because it is a single-joint muscle, whereas the GA is biarticular and acts at both the ankle and knee joints. This allowed us to provide stimulation specifically related to ankle joint control without confounding influences from knee motion. Each electrode pair was connected to a channel of the function generator (SEN-8203, Nihon Kohden) through an isolator (SS104-J, Nihon Kohden). The function generator was responsible for delivering electrical stimulation signals to the electrodes. A microcontroller (Arduino Uno, Arduino) controlled the stimulation process by sending trigger signals to the function generator based on the real-time motion data from the marker placed at the front edge of the balance board through the motion capture system. The motion capture system transmitted the motion data to the microcontroller at 100 Hz, which processed the height of the balance-board marker (*h*) as the control parameter to determine the trigger signals. The EMS stimulation was applied to the TA or SO based on two predefined height thresholds of the balance board ($th_{TA}$, $th_{SO}$), as illustrated in Fig. 3. When the height was below $th_{TA}$, the microcontroller sent a trigger signal to activate EMS on the TA, and when it was above $th_{SO}$, a trigger signal was sent to activate EMS on the SO. In both cases, EMS was delivered at a fixed stimulation intensity and was not modulated according to the magnitude of the board tilt. When the height was between $th_{TA}$ and $th_{SO}$, EMS was turned off. The thresholds $th_{TA}$ and $th_{SO}$ were determined in advance from the results of the BB task in the pre-test, as

1 $$ th_{{TA}} = h_{{mean}} - h_{{std}} ,\,\,th_{{SO}} = h_{{mean}} + h_{{std}} , $$

where $h_{mean}$ is the average height of the marker recorded for 60 s (20 s *× * 3 trials; the first and final 5 s were excluded), and $h_{std}$ is the standard deviation of the marker height values. This stimulation pattern assisted ankle movements that helped return the balance board toward a horizontal position.

Between the pre-test and training test, the stimulus intensity was individually calibrated for each participant. The EMS output was gradually increased until involuntary ankle dorsiflexion or plantarflexion was observed, ensuring that the stimulation remained within a pain-free range. Based on previous studies in the context of motor learning using EMS-assisted joint movements [20–22, 41], the pulse width was set to 200 µs, the frequency to 200 Hz, and the maximum current to 20 mA. The relatively high frequency was chosen to provide smooth, continuous ankle assistance without pulsatile contractions that could interfere with fine balance adjustments during dynamic board tilts. Before the training session, each participant stood on the board while holding onto the parallel bars and tilted the board forward and backward to feel the stimulation. The stimulation intensity was then fine-tuned to ensure that the subjective perception of EMS intensity was consistent across all stimulation sites, while providing movement assistance without causing excessive muscle contractions. During this period, participants were informed that when the board tilted beyond a certain level, stimulation-induced muscle contractions would assist in returning the board to a horizontal position, and they were asked to voluntarily move their ankles in accordance with the assisted movement.

The training test lasted for five minutes. Just before the start, participants placed both feet on the designated positions on the board and held onto the parallel bars while waiting. Upon the signal, they released their hands, crossed their arms in front of the chest, and began the practice. The standing position, basic posture, and instructions during practice were the same as in the BB tasks in the pre- and post-tests. To minimize the effects of fatigue, participants took breaks as needed. During these breaks, they returned to the waiting posture used just before the start. No participants reported fatigue or pain during or after the 5-minute training session.

### Analysis

#### Data processing

All raw data from the pre- and post-tests were processed using MATLAB 2023a. To eliminate transient instability, the initial and final 2 s of each trial were removed. For the ST task, COP was recorded at 1 kHz using a force plate, which is considered the gold standard for evaluating postural sway [42, 43]. The COP signals in the mediolateral (ML) and anteroposterior (AP) directions were low-pass filtered using a 4th-order zero-lag Butterworth filter with a cut-off frequency of 8 Hz [31]. For both ST and BB tasks, the tri-axial positions of markers attached to ten body locations were measured using an infrared motion capture system (Vicon, Vicon Motion Systems) with a sampling rate of 100 Hz. In the BB task, additional markers were placed on the balance board (three locations), and COP data were not recorded due to the absence of a force plate. Marker trajectories were low-pass filtered using a 4th-order zero-lag Butterworth filter with a 10-Hz cut-off. For each bilateral body region (shoulders, pelvis, knees, ankles, and toes), the marker with fewer missing frames was selected as the representative for that region. EMG signals were collected at 1 kHz from four muscle sites on the right lower limb in both tasks. The EMG data were band-pass filtered using a 4th-order zero-lag Butterworth filter with a frequency range of 20–450 Hz to remove movement artifacts and high-frequency noise.

To minimize potential subjective bias in the data analysis, the correspondence between participant IDs and their respective groups was blinded to all co-authors except the corresponding author conducting the experiment.

#### Overview of outcomes

To comprehensively evaluate the training effects, outcomes were categorized into three hierarchical levels as follows:

- Sensory contribution: To assess the impact of balance training on sensory reweighting, both linear and nonlinear analyses of COP trajectories during the ST task were conducted. In the linear analysis, COP sway was calculated under each standing condition to examine the influence of training on the sensory systems. In the nonlinear analysis, frequency analysis of the COP signals was performed to evaluate the contributions of different sensory modalities to postural control.
- Task performance: To evaluate the training effects on balance board control, the balance board sway (BB sway) was analyzed in the BB task, as the primary goal of the task was to control the board to remain level. In this task, a smaller BB sway (i.e., lower velocity and RMS) indicates superior task performance, since participants were instructed to maintain the balance board in a level position with minimal oscillation.
- Postural control strategies: To evaluate changes in the underlying motor processes, measures including body segment sway, joint angle sway, sample entropy, cross-correlation coefficient (CCR), and co-contraction index (CCI) were analyzed for both tasks. Unlike performance measures, these are interpreted collectively to understand postural control strategies. For example, a shift toward a hip-dominant strategy can be identified by increased pelvic oscillations in conjunction with a lower CCI of the hip muscles, reflecting a more flexible and adaptive use of the proximal segments.

#### Linear and nonlinear analysis of COP data

Postural stability in the ST task was quantified from the COP trajectories recorded by the force plate. For each trial, the mean velocity and root-mean-square (RMS) of the COP were calculated in both the ML and AP directions. We also performed a nonlinear frequency analysis of the COP time series in each direction, since COP behavior reflects the sensory contribution of the visual, vestibular, and proprioceptive systems. After removing the direct-current component, the power spectral density was obtained via the Fourier transform, and the integrated power was computed within three frequency bands: 0−0.3 Hz, 0.3–1 Hz, and 1–3 Hz. These bands have been previously associated with reliance on visual (0−0.3 Hz), vestibular (0.3–1 Hz), and proprioceptive (1–3 Hz) sensory inputs, as suggested in earlier studies [27–31]. This analysis is considered to more clearly reflect sensory contributions under static standing conditions; however, under more challenging postural conditions, the results may be influenced by non-sensory factors such as muscular responses and motor strategies, making interpretation more difficult. Therefore, the values calculated under the eyes open (EO) condition were used for pre- and post-training comparisons.

#### Calculation of marker sway using motion capture data

To examine the effects of training on board stability in the BB task, the mean velocity and RMS of the BB sway were calculated in the vertical direction using a marker placed at the front of the board. If the front marker became detached and fell off, a marker at the rear of the board was used as a substitute.

To better understand how each training modality influences the control of individual body segments, we analyzed body segment sway in both the ST and BB tasks. The mean velocity and RMS in the ML and AP directions were computed for representative markers located at the shoulder, pelvis, and knee.

To assess changes in joint control strategies, joint angle sway in the sagittal plane was calculated for both the ST and BB tasks. The ankle angle was defined as the angle formed in the sagittal plane by markers placed on the knee, ankle, and toe; the knee angle was derived from markers on the pelvis, knee, and ankle; and the hip angle was calculated using markers on the shoulder, pelvis, and knee. The mean velocity and RMS were then calculated using the time-series data of each joint angle.

#### Sample entropy

To quantify the complexity and regularity of body sway signals during postural control, we utilized sample entropy (SampEn) as proposed by Richman and Moorman [44]. SampEn evaluates the likelihood that similar patterns of a given length *m* will remain similar when one additional point is added, thereby capturing the complexity or randomness of the time series. A higher SampEn value indicates greater irregularity and complexity in the signal.

In the ST task, the time series COP data in the AP and ML directions, which were originally sampled at 1000 Hz, were downsampled to 200 Hz by averaging every five consecutive points [45]. In the BB task, the time-series data of the BB angle (defined as the angle between the line connecting the anterior and posterior markers of the BB and the horizontal plane of the laboratory coordinate system in the sagittal plane) were utilized. SampEn was then calculated under the following conditions. The template length *m* was set to 2. The similarity threshold *r*, which defines how closely two patterns must match to be considered similar, was expressed as a proportion of the signal’s standard deviation (SD) and was fixed at 0.2 in this study. Distances between templates were quantified using the Chebyshev metric, which computes the maximum absolute difference between corresponding elements of the patterns. For each template and threshold combination, the number of matched pattern pairs within the threshold distance was counted as B, and the number of matched pairs that remained similar when extended by one point was counted as A. SampEn was then computed by

2 $$\begin{aligned} \textrm{SampEn}(m, r) = -\log \left( \frac{A}{B} \right) . \end{aligned}$$

In cases where either A or B was zero, leading to an undefined or infinite SampEn value, we substituted a finite maximum value based on the minimum non-zero conditional probability, defined as $\frac{A}{B} = \frac{2}{(N - m - 1)(N - m)}$, where *N* is the length of the time series.

#### CCR

The cross-correlation coefficient (CCR) quantifies how strongly two time series are interdependent or alike. For two signals, *x*(*t*) and *y(t+Δ t)*, the Pearson correlation coefficient is calculated while incrementally shifting one series relative to the other by a time lag *Δ t*. The CCR at a given *Δ t* equals 1 when the two waveforms coincide perfectly, –1 when they coincide after a sign inversion, and 0 when no linear similarity exists. In the present study, CCR was computed for every trial of both the BB and ST tasks. In the ST task, the analysis used the time-series data of the anteroposterior COP signal (down-sampled to 100 Hz), ankle, knee, and hip joint angles, as joint kinematics were analyzed only in the sagittal plane. In the BB task, the analysis used the time-series data of the BB angle, ankle, knee, and hip joint angles. For each pair of variables, the absolute value of the CCR was obtained across all lags, and both the maximum absolute CCR and its corresponding lag were used as representative measures for statistical comparison.

#### Muscle CCI

To investigate changes in joint-level neuromuscular strategies through training, we computed the co-contraction index (CCI) of ankle and hip muscles during both the BB and ST tasks using EMG signals. The CCI quantifies the degree of simultaneous activation between an agonist–antagonist muscle. The filtered EMG data of each muscle were rectified, and the RMS was calculated over a 100-ms sliding window. For the ankle joint, the TA and GA were defined as the agonist–antagonist pair; for the hip joint, the RF and HA were used. The CCI for each joint was calculated using the following formulas [46].

3 $$\begin{aligned} \textrm{CCI}_{\textrm{ankle}} = \frac{2 \cdot \sum _{t} \min \left( \textrm{RMS}_{\textrm{TA}}(t), \textrm{RMS}_{\textrm{GA}}(t)\right) }{\sum _{t} \max \left( \textrm{RMS}_{\textrm{TA}}(t), \textrm{RMS}_{\textrm{GA}}(t)\right) + \sum _{t} \min \left( \textrm{RMS}_{\textrm{TA}}(t), \textrm{RMS}_{\textrm{GA}}(t)\right) } \times 100. \end{aligned}$$

4 $$\begin{aligned} \textrm{CCI}_{\textrm{hip}} = \frac{2 \cdot \sum _{t} \min \left( \textrm{RMS}_{\textrm{RF}}(t), \textrm{RMS}_{\textrm{HA}}(t)\right) }{\sum _{t} \max \left( \textrm{RMS}_{\textrm{RF}}(t), \textrm{RMS}_{\textrm{HA}}(t)\right) + \sum _{t} \min \left( \textrm{RMS}_{\textrm{RF}}(t), \textrm{RMS}_{\textrm{HA}}(t)\right) } \times 100. \end{aligned}$$

Here, $\textrm{RMS}_{\textrm{TA}}(t)$, $\textrm{RMS}_{\textrm{GA}}(t)$, $\textrm{RMS}_{\textrm{RF}}(t)$, and $\textrm{RMS}_{\textrm{HA}}(t)$ represent the RMS values of the corresponding muscles at time *t*. The numerator reflects the overlapping activation of the muscle pair, while the denominator normalizes this overlap by the total activation level. The resulting CCI value ranges from 0 to 100, where higher values indicate stronger co-contraction (i.e., a joint-stiffness strategy), and lower values indicate weaker co-contraction (i.e., a more flexible, joint-relaxing strategy).

#### Statistics

To ensure that the training load was consistent across participants, the actual total practice time in the training session—defined as the total duration of active balance board standing, excluding periods where participants held the handrails for rest as determined by visual inspection of video recordings—was compared between the EMS and control groups using an unpaired *t*-test. For each postural control measure, the average of three trials was used as the representative value for each participant in the pre- and post-tests. To assess the normality of the data, the Shapiro–Wilk test was performed on each group’s pre- and post-test data series. If all data series met the assumption of normality, a two-way mixed-design repeated-measures analysis of variance (ANOVA) was conducted, with one between-group factor (group: EMS vs. control) and one within-group factor (test session: pre vs. post), to examine group differences in the analyzed values. If a significant interaction was observed, a Bonferroni-corrected paired *t*-test was utilized for post hoc comparisons. If the assumption of normality was not satisfied for any data series, non-parametric tests were applied: the Wilcoxon signed-rank test was used for within-group comparisons (pre vs. post), and the Wilcoxon rank-sum test was used for between-group comparisons (EMS vs. control) at each of pre- and post-tests. Bonferroni correction was applied to all post-hoc statistical comparisons. Test statistics were reported as *F* values for analyses of ANOVA, *t* values for parametric *t*-tests, and *Z* values for non-parametric tests. Effect sizes were calculated for all comparisons (Cohen’s *d* for parametric data and *r* for non-parametric data) [47]. The level of statistical significance was set to *p < 0.05*.

## Results

### Total practice time

The actual total practice time in the training session was 3 min 52 s ± 42 s in the EMS group and 3 min 30 s ± 43 s in the control group. An unpaired *t*-test revealed no significant difference between the groups ($t(\text {37}) = 1.626, p = 0.112$; n = 39 due to one recording failure). This confirms that the total duration of active training was comparable between the two groups.

### COP sway

**Fig. 4:**
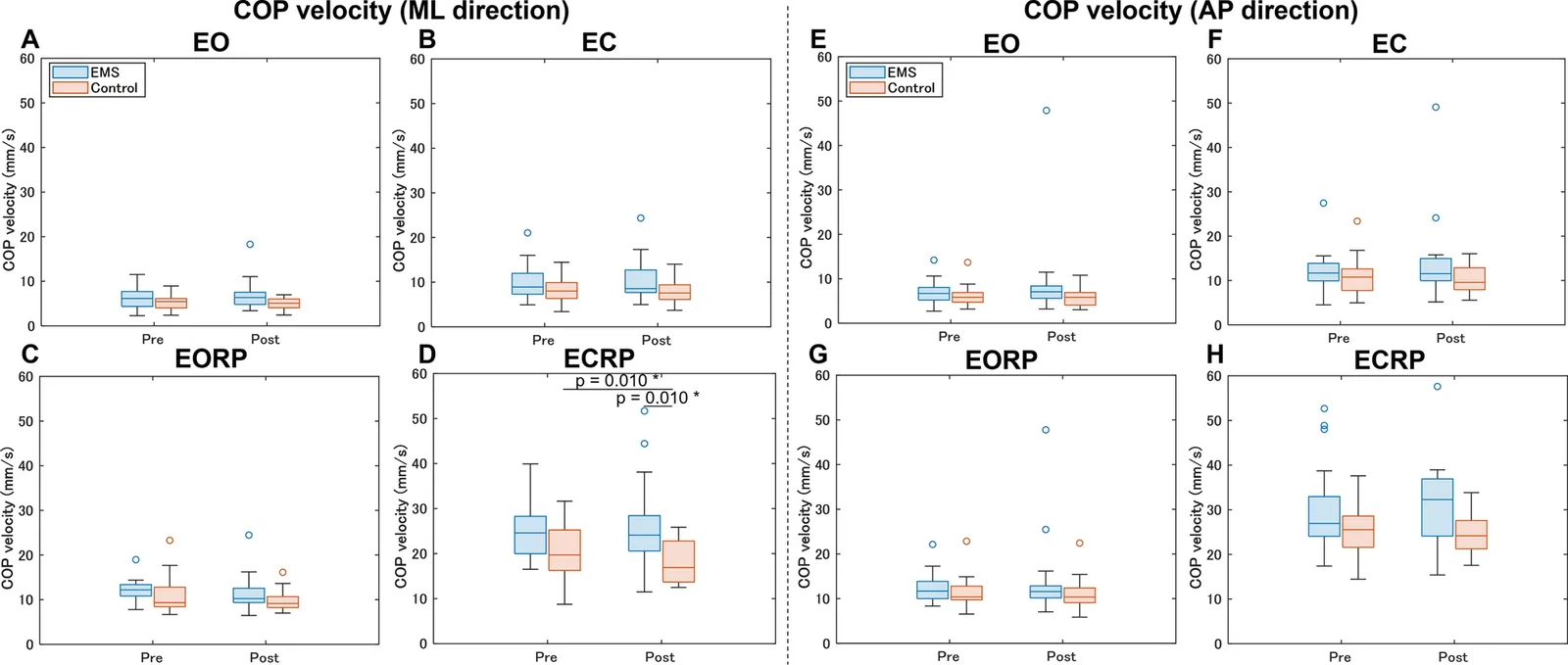
Box plots of COP velocity in the AP and ML directions between pre- and post-tests for EMS and control groups. The horizontal line within the box indicates the median, and the bottom and top edges of the box indicate the 25th and 75th percentiles, respectively. Plots outside the box represent outliers. P-values are shown for statistically significant comparisons. *COP* center of pressure, *ML* mediolateral, *AP* anteroposterior, *EO *eyes open, *EC* eyes closed, *EORP* eyes open on rubber pad, *ECRP* eyes closed on foam rubber pad. For visualization purposes, outliers far beyond the interquartile range may fall outside the plot in all graphs

**Fig. 5:**
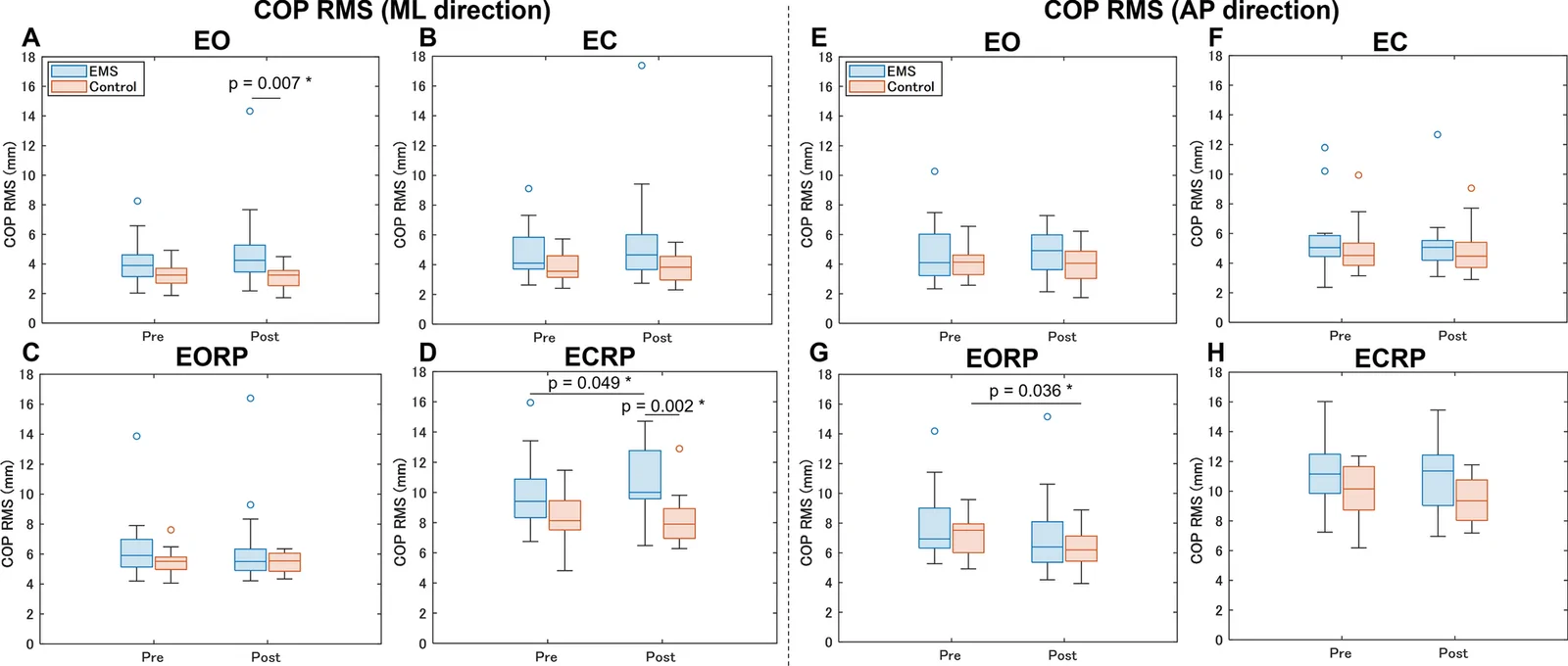
Box plots of COP RMS in the AP and ML directions between pre- and post-tests for EMS and control groups. P-values are shown for statistically significant comparisons. *COP* center of pressure, *ML* mediolateral, *AP* anteroposterior, *EO * eyes open, *EC* eyes closed, *EORP* eyes open on rubber pad, *ECRP* eyes closed on foam rubber pad

In the COP velocity results (Fig. 4), while there was no significant difference in the AP direction, significant differences were observed in the ML direction under the ECRP condition. Specifically, the control group showed a significant decrease from pre- to post-test (*Z = 3.024*, *p = 0.010*, *r = 0.676*; Fig. 4D), and in the post-test, the EMS group exhibited significantly greater sway than the control group (*Z = 3.016*, *p = 0.010*, *r = 0.477*; Fig. 4D). For COP RMS (Fig. 5), in the ML direction during the post-test, the EMS group demonstrated significantly larger sway than the control group under both the EO (*Z = 3.124*, *p = 0.007*, *r = 0.494*; Fig. 5A) and ECRP (*Z = 3.476*, *p = 0.002*, *r = 0.550*; Fig. 5D) conditions. Additionally, under the ECRP condition, the EMS group in the post-test showed significantly greater sway than in the pre-test (*Z = 2.501*, *p = 0.049*, *r = 0.559*; Fig. 5D). In the AP direction, a significant pre-to-post reduction in sway was also observed in the control group under the EORP condition (*Z = 2.613*, *p = 0.036*, *r = 0.584*; Fig. 5G).

### Power spectral density

**Fig. 6:**
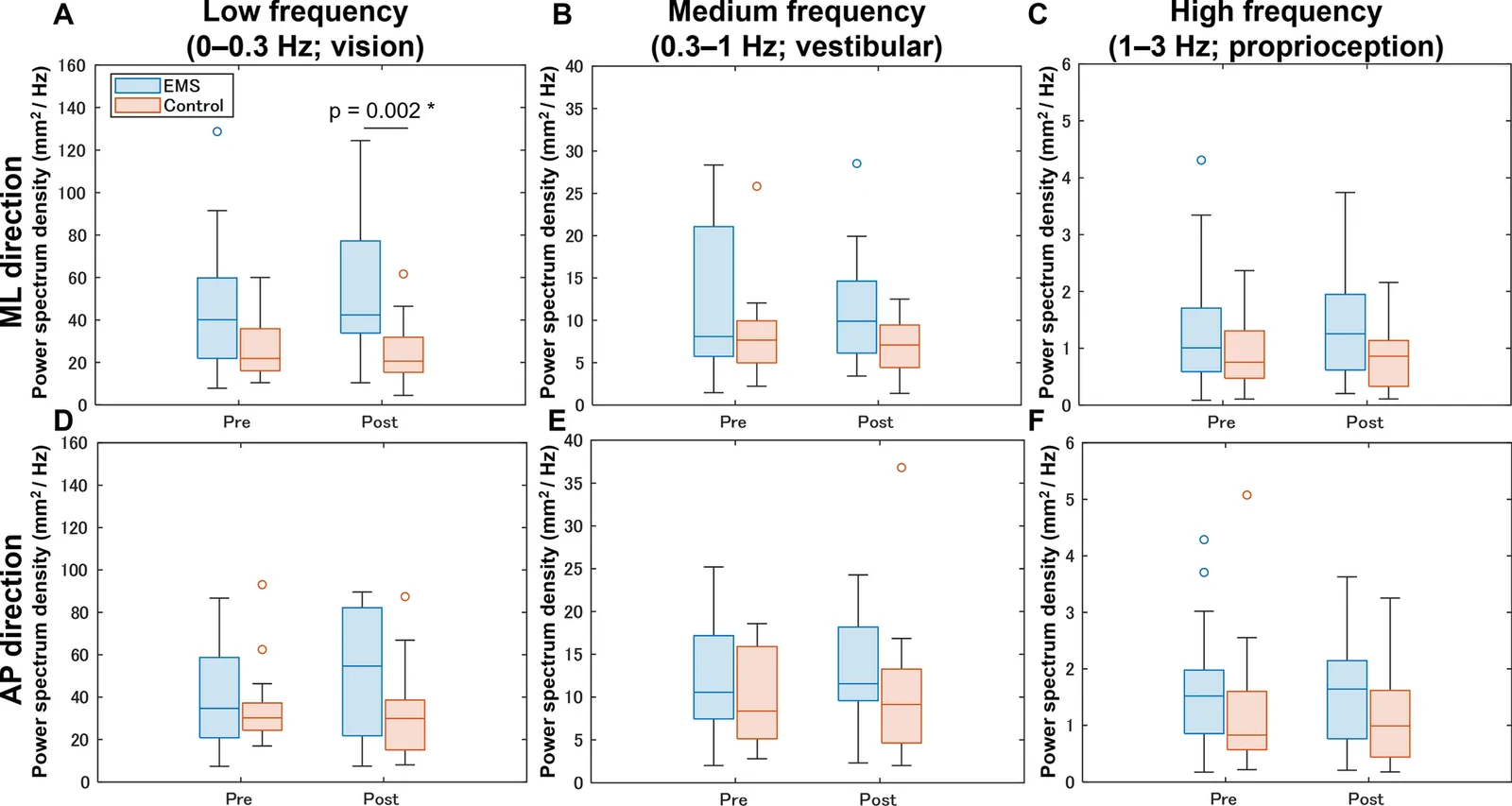
Box plots of power spectral density of the three frequency bands in the AP and ML directions between pre- and post-tests for EMS and control groups. Each frequency band reflects the contribution of a specific sensory system to postural control: low (0-−0.3 Hz; vision), medium (0.3–1 Hz; vestibular), and high (1–3 Hz; proprioception). Note that the vertical axis scale differs across frequency ranges to improve the visibility. P-values are shown for statistically significant comparisons

Figure 6 shows the box plots of power spectral density in three frequency bands between pre- and post-tests for the EMS and control groups. The results of the non-parametric tests showed that, in the low-frequency band of ML direction for the post-test, the EMS group exhibited significantly higher values compared to the control group (*Z = 3.449*, *p = 0.002*, *r = 0.545*; Fig. 6A). In contrast, no significant differences were observed in the medium- and high-frequency bands.

### BB and body segment sway

**Fig. 7:**
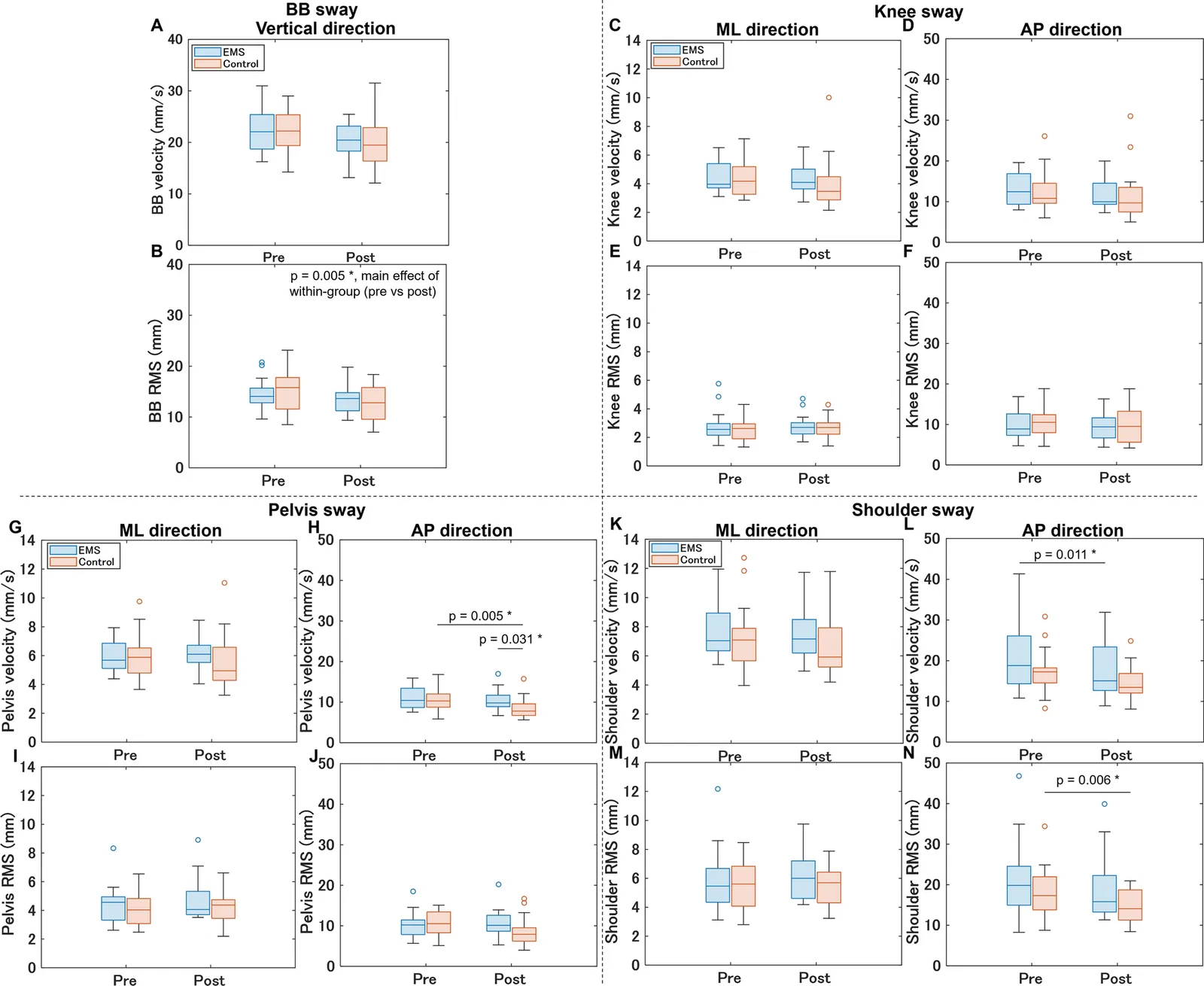
Box plots of BB and body segment sway during the BB task in the pre- and post-tests for each group. The graphs in each segment depict velocity (upper side) and RMS (lower side). Note that the ML and AP directions use different vertical scales. P-values indicate statistically significant within- or between-group comparisons, while labels above the plots indicate significant main effects of within-group (pre vs. post)

In the BB sway velocity (Fig. 7A), no significant differences were found in the non-parametric test results. In the BB sway RMS, which was analyzed using a parametric test, there was a significant main effect for the within-group factor ($F_{1, 38} = 8.926$, *p = 0.005*; Fig. 7B), but no significant interaction was found between the two factors. For knee sway (Fig. 7C–F), no significant differences were observed in either group. Regarding pelvis sway, the control group showed a significant decrease in AP velocity from pre- to post-test (*Z = 3.248*, *p = 0.005*, *r = 0.726*; Fig. 7H), and in the post-test, the EMS group exhibited significantly greater AP velocity than the control group (*Z = 2.664*, *p = 0.031*, *r = 0.421*; Fig. 7H). For shoulder sway, the EMS group demonstrated a significant decrease in AP velocity from pre- to post-test (*Z = 2.987*, *p = 0.011*, *r = 0.668*; Fig. 7L), while the control group showed a significant decrease in AP RMS from pre- to post-test (*Z = 3.173*, *p = 0.006*, *r = 0.710*; Fig. 7N).

### Joint angle sway

**Fig. 8:**
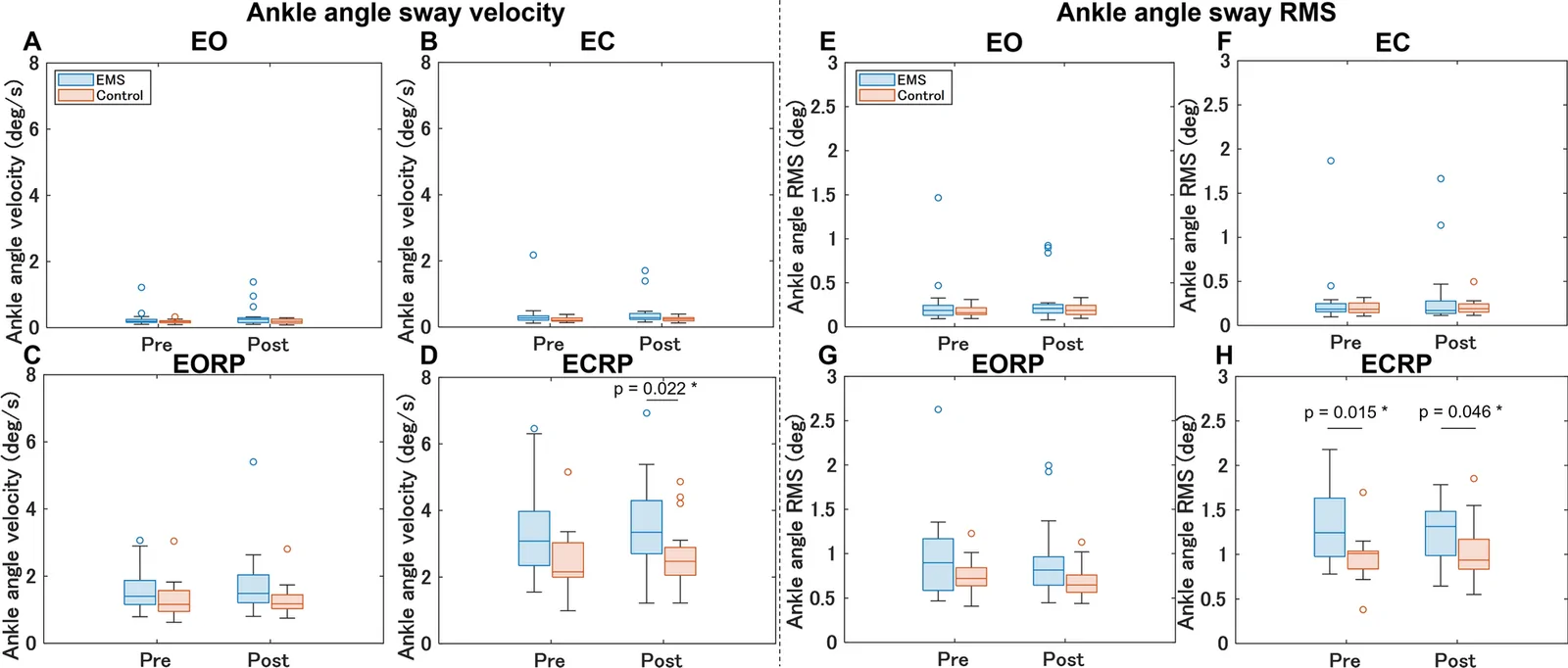
Box plots of ankle angle sway during the ST task under each standing condition in the pre- and post-tests for each group. P-values indicate statistically significant within- or between-group comparisons

**Fig. 9:**
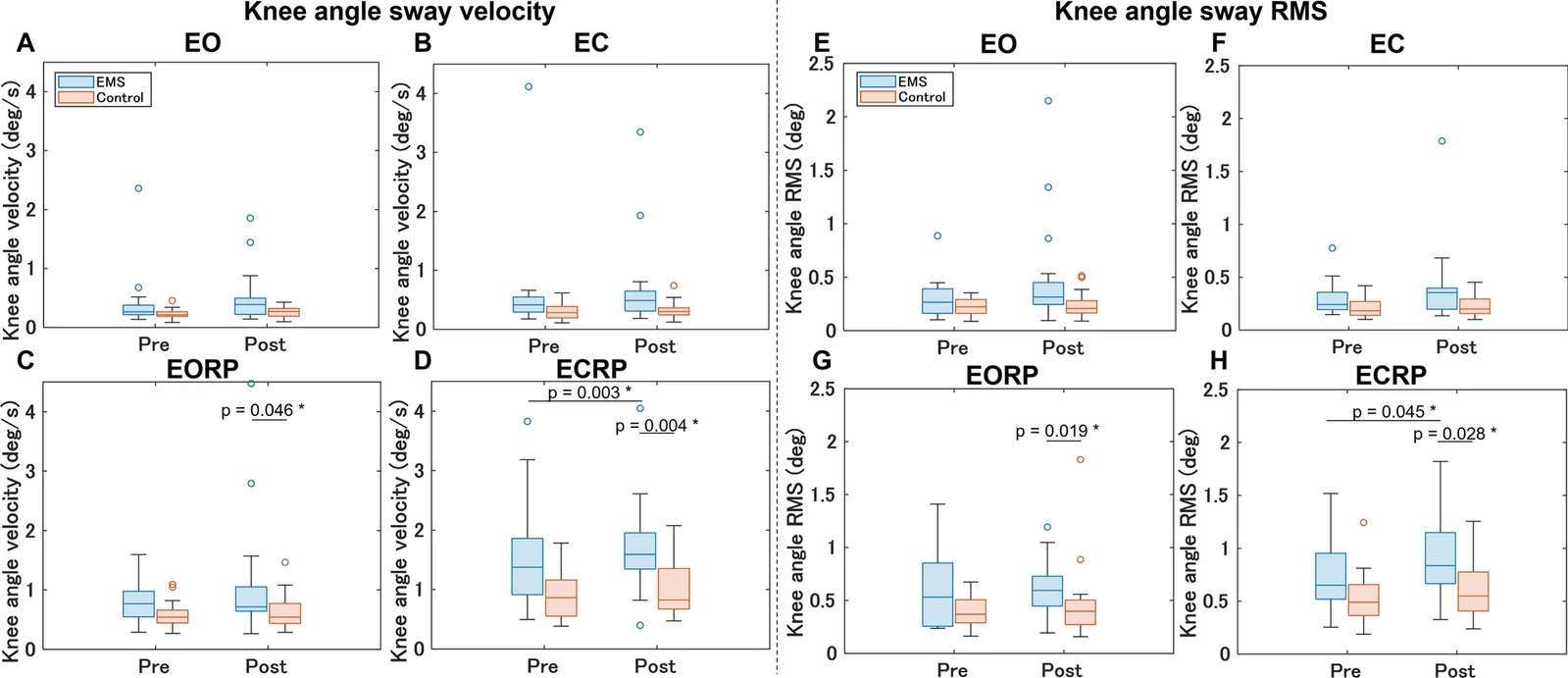
Box plots of knee angle sway during the ST task under each standing condition in the pre- and post-tests for each group

**Fig. 10:**
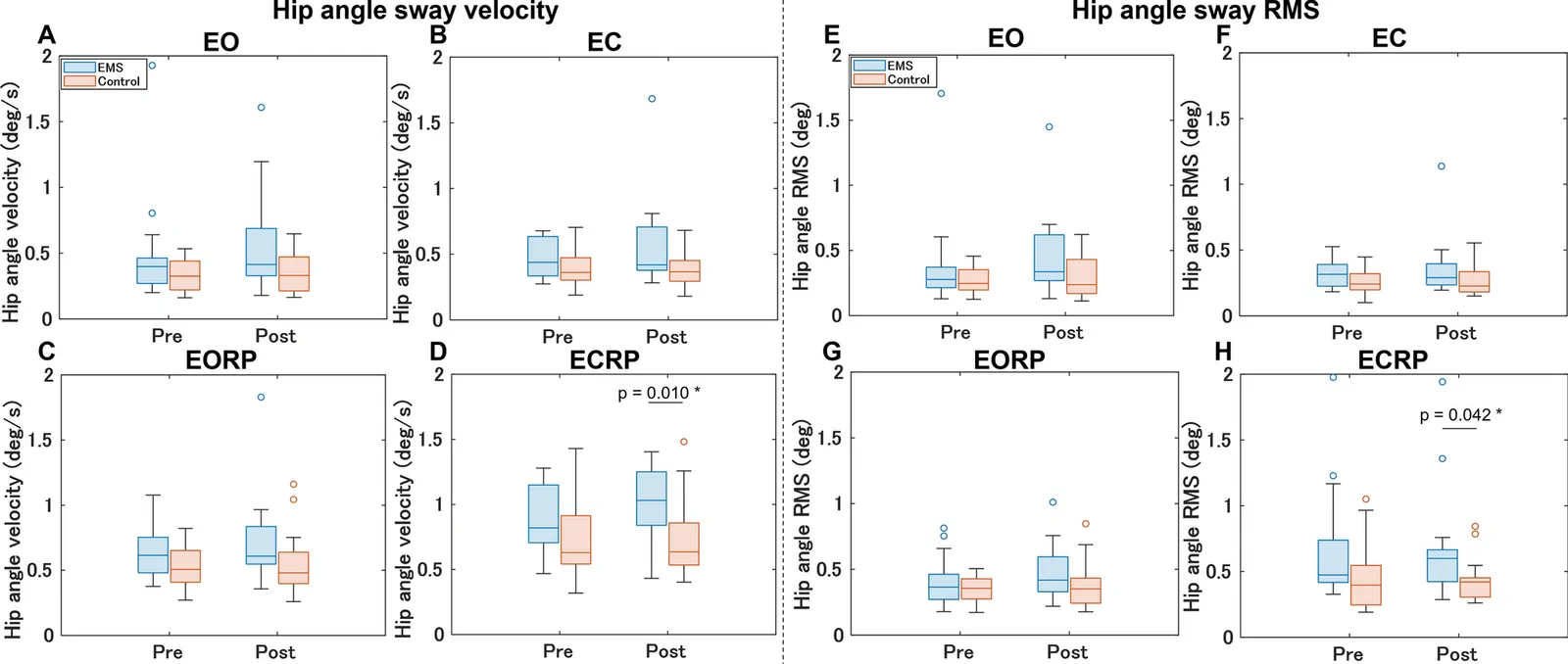
Box plots of hip angle sway during the ST task under each standing condition in the pre- and post-tests for each group

Figures 8, 9, 10 present box plots of ankle, knee, and hip joint angle sway, respectively, under each standing condition of the ST task for both groups during the pre- and post-tests. For ankle joint angles, under the ECRP condition, the EMS group showed significantly greater sway velocity in the post-test compared to the control group (*Z = 2.773*, *p = 0.022*, *r = 0.438* for velocity; Fig. 8D, *Z = 2.529*, *p = 0.046*, *r = 0.400* for RMS; Fig. 8H), and the EMS group also showed significantly greater RMS in the pre-test under the same condition (*Z = 2.908*, *p = 0.015*, *r = 0.460*; Fig. 8H). For knee joint angles, under the ECRP condition, both sway velocity and RMS increased from pre- to post-test in the EMS group (*Z = 3.397*, *p = 0.003*, *r = 0.760* for velocity; Fig. 9D, *Z = 2.539*, *p = 0.045*, *r = 0.568* for RMS; Fig. 9H), and the EMS group showed significantly greater sway in the post-test compared to the control group (*Z = 3.287*, *p = 0.004*, *r = 0.520* for velocity; Fig. 9D, *Z = 2.691*, *p = 0.028*, *r = 0.426* for RMS; Fig. 9H). Additionally, under the EORP condition, both velocity and RMS in the post-test were significantly greater in the EMS group than in the control group (*Z = 2.529*, *p = 0.046*, *r = 0.400* for velocity; Fig. 9C, *Z = 2.827*, *p = 0.019*, *r = 0.447* for RMS; Fig. 9G). For hip joint angles, the EMS group showed significantly greater sway velocity and RMS in the pre-test under the ECRP condition (*Z = 3.016*, *p = 0.010*, *r = 0.477* for velocity; Fig. 10D, *Z = 2.556*, *p = 0.042*, *r = 0.404* for RMS; Fig. 10H).

**Fig. 11:**
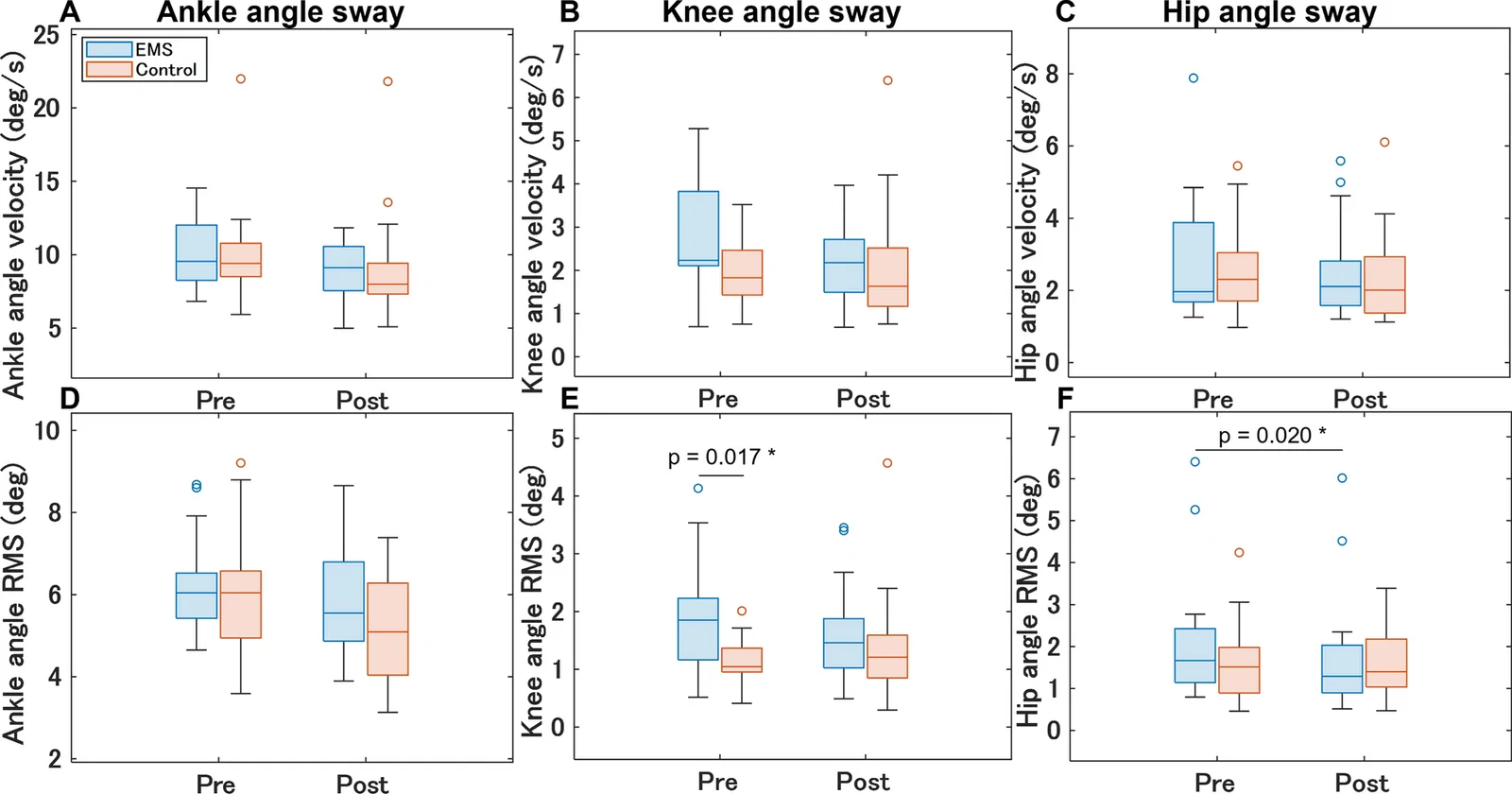
Box plots of ankle, knee, and hip angle sway during the BB task in the pre- and post-tests for each group

In the BB task, as shown in Fig. 11, the RMS of the hip joint angle in the EMS group significantly decreased after training (*Z = 2.800*, *p = 0.020*, *r = 0.626*; Fig. 11F). In the pre-test, the EMS group showed significantly greater RMS in knee joint angles than the control group (*Z = 2.854*, *p = 0.017*, *r = 0.451*; Fig. 11E).

### Sample entropy

**Fig. 12:**
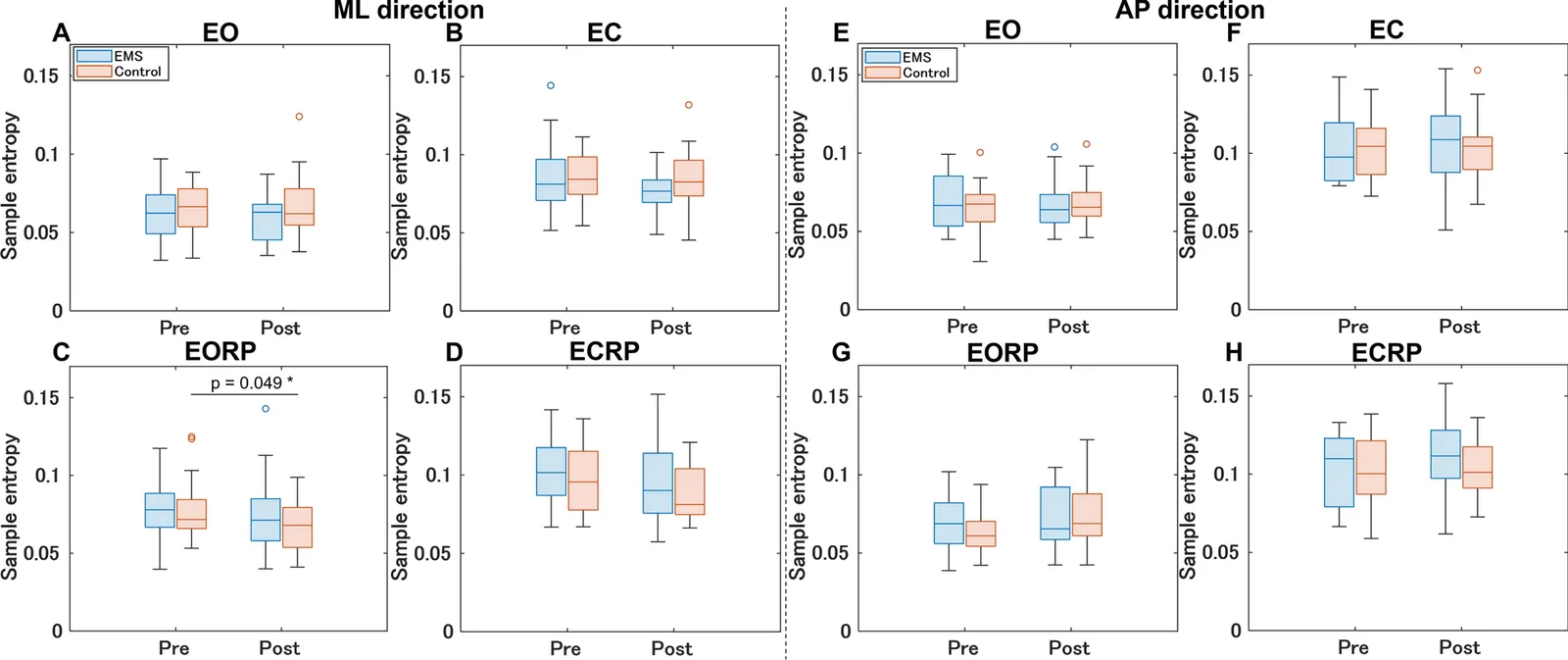
Box plots of sample entropy during the ST task under each standing condition in the pre- and post-tests for each group

Significant changes in sample entropy were observed only in the ST task. Specifically, in the ML direction under the EORP condition, the control group significantly decreased from pre- to post-test (*Z = 2.501*, *p = 0.049*, *r = 0.559*; Fig. 12C), but there was no significant difference in the AP direction. This indicates a reduction of the complexity during postural control after training.

### CCR

**Fig. 13:**
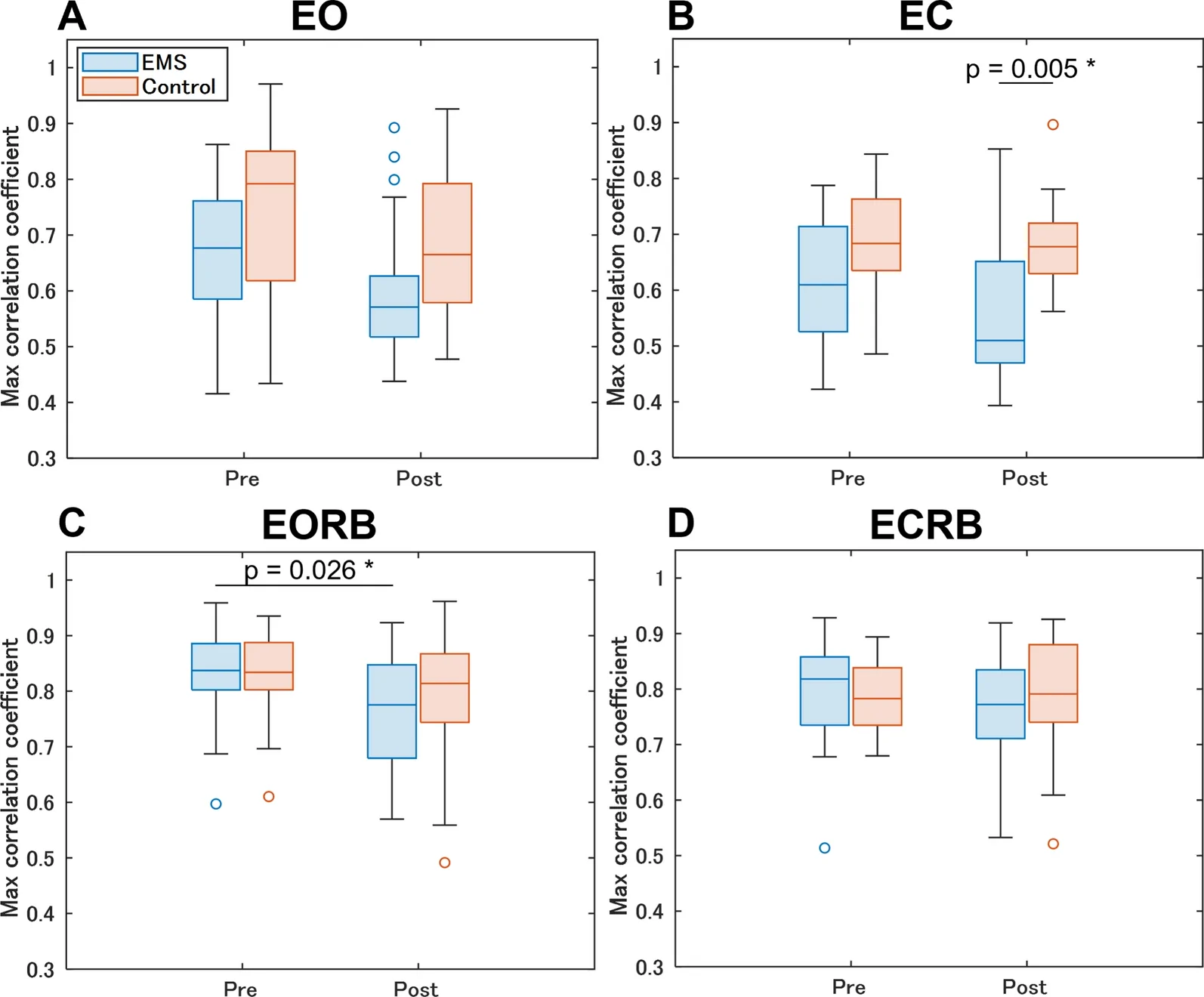
Box plots of CCR between AP COP and ankle angle during the ST task under each standing condition in the pre- and post-tests for each group

Figure 13 shows box plots of the CCR between AP COP and ankle angle during the ST task under each standing condition in the pre- and post-tests for each group. In the post-test under the EC condition, the EMS group showed a significantly lower maximum correlation coefficient in CCR compared to the control group (*Z = 3.232*, *p = 0.005*, *r = 0.511*; Fig. 13B). In addition, within the EMS group, the maximum correlation coefficient significantly decreased from pre- to post-test under the EORP condition (*Z = 2.725*, *p = 0.026*, *r = 0.609*; Fig. 13C). The CCR results indicate a decreasing trend in the coupling between COP in the AP direction and ankle joint angle in the EMS group after training.

### Muscle CCI

**Fig. 14:**
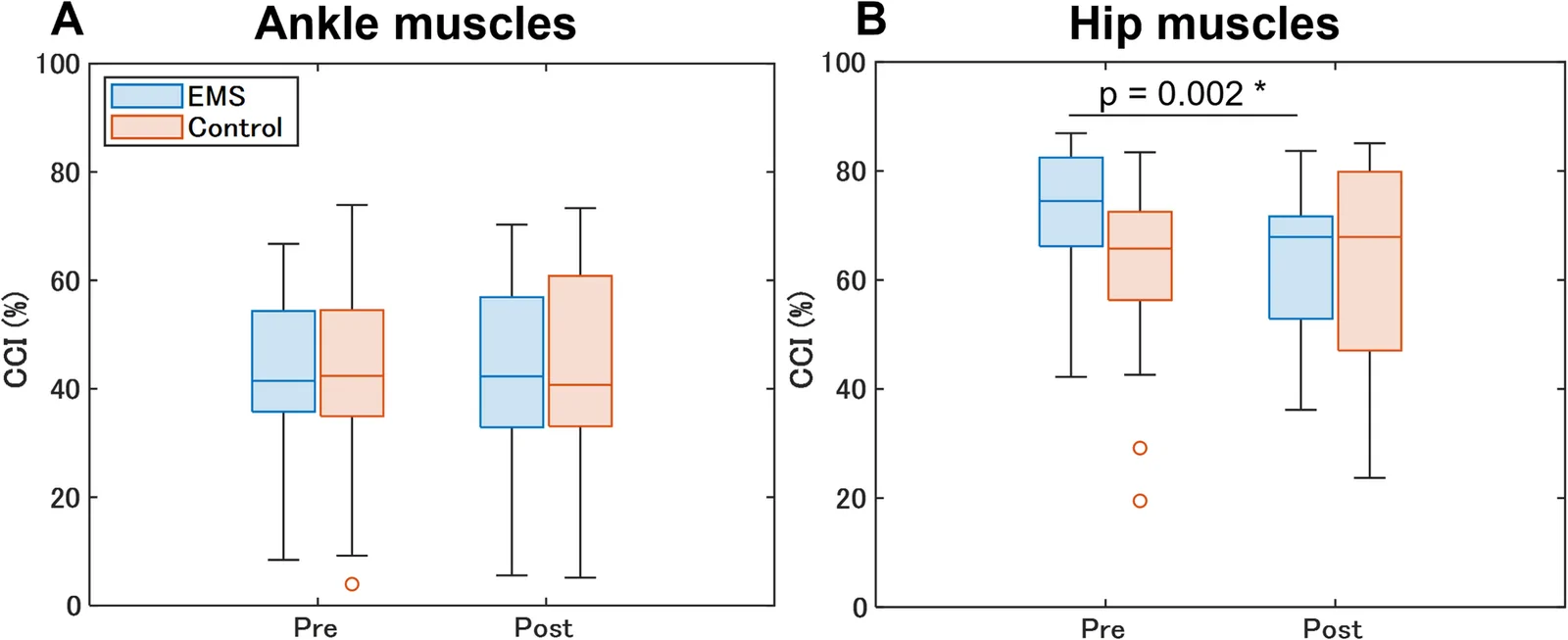
Box plots of CCI in ankle and hip muscles during the BB task in the pre- and post-tests for each group

As shown in Fig. 14, although no significant differences were observed in the ankle muscle CCI during the BB task, the hip muscle CCI significantly decreased from pre- to post-test in the EMS group (*Z = 3.472*, *p = 0.002*, *r = 0.776*; Fig. 14B).

## Discussion

### Effects of EMS-based balance training on the ST task

To examine how EMS-based balance training influences sensory reweighting during quiet standing, we analyzed the frequency characteristics of COP sway. Time frequency analysis of the COP (Fig. 6) revealed that the PSD in the low-frequency band of ML COP sway was significantly higher in the EMS group compared to the control group in the post-test. Since low-frequency components of COP sway are thought to reflect visual contributions to postural control [27–31], this result suggests that participants controlled standing posture with greater reliance on vision (up-weighted visual weight) following EMS training. This interpretation is further supported by the COP sway results (Figs. 4 and 5): the EMS group exhibited greater sway after training compared to the control group under the ECRP condition, indicating less stable postural control when visual and somatosensory inputs are limited or unreliable. Because this condition reduces the reliability of both visual and somatosensory cues from the feet, vestibular information becomes critical for stabilization [8, 48, 49]. Thus, the increased sway in the EMS group indicates that EMS assistance promoted a shift toward greater visual reliance. This finding substantiates at the group level our previous research using a similar EMS-based balance-board task [18], which demonstrated that individuals who showed greater stabilization during EMS presentation exhibited an increase in visual weight after the task. Compared with that study, the actual total practice time in the training session was approximately 2.5 times longer (about 3.7 min excluding breaks, versus 1.5 min previously), which may have facilitated immediate adaptation to EMS. Overall, these findings support the hypothesis that EMS-based balance training facilitates visual reweighting in postural control.

To investigate how EMS-based balance training affects static postural control strategies during quiet standing, we further examined COP complexity and joint coordination patterns. The control group showed a decreased sample entropy in the EORP condition from pre- to post-training (Fig. 12), indicating that balance board training without EMS led to a reduction in the complexity of COP dynamics. Joint angle sway (Figs. 8, 9, 10) increased after EMS training across all joints under unstable surface conditions (EORP, ECRP), indicating that the EMS group transitioned to a control pattern involving greater movement across multiple joints. Furthermore, the CCR results (Fig. 13) demonstrated that the maximum correlation coefficient between COP and ankle joint angle decreased after training under the EC and EORP conditions in the EMS group. Considering that the correlation between the COP and ankle angle approaches 1 in a typical inverted pendulum model, the observed decrease in this correlation reflects a decoupling of ankle motion from COP, suggesting that proximal joints became more prominent. Integrating the results of these indicators related to postural control strategy, it is suggested that postural control in the EMS group after practice was no longer solely governed by the ankle joint but instead reflected the involvement of other joints, indicating a shift toward a more complex, multi-joint coordination strategy.

Taken together, the ST task results indicate that EMS-based balance training was associated with both a shift in sensory weighting toward greater visual reliance and a transition from ankle-dominant to more multi-joint coordination during quiet standing.

### Effects of EMS-based balance training on the BB task

To examine how EMS-based balance training influences postural control strategies during an active balance task, we analyzed body segment kinematics, joint motion, and muscle coordination during the BB task. In this task, participants aimed to keep a board that tilted forward and backward in a horizontal position; therefore, task performance was reflected in the vertical sway of the marker at the edge of the board. As shown in Fig. 7A and B, a significant main effect of within-group factor (test session: pre vs. post) was observed for the board sway RMS, indicating that task performance (i.e., stabilization of the balance board) improved regardless of EMS. However, no significant group differences were confirmed. This indicates that the 5-minute training produced comparable improvements in overall task performance across groups.

Interestingly, however, differences emerged in indicators related to postural control strategies. The AP sway velocity of each body segment (Fig. 7) revealed distinct adaptations between groups. In the EMS group, shoulder sway velocity decreased after training (Fig. 7L), whereas pelvic sway velocity did not change and remained larger than that of the control group at post-test (Fig. 7H). In contrast, the control group showed a decrease in pelvic sway velocity after training (Fig. 7H) while shoulder sway velocity remained unchanged. Furthermore, RMS of hip joint angle sway in the AP direction decreased after training in the EMS group, although its velocity did not change (Fig. 11F), suggesting that hip motion was regulated within a narrower range while maintaining movement speed. These kinematic results suggest that the EMS group stabilized the upper body while maintaining pelvic motion, thereby utilizing relative pelvic motion for postural regulation. In contrast, the control group appeared to stabilize posture by reducing pelvic motion while maintaining shoulder movement, indicating a control strategy relying more on relative upper-body motion. Regarding CCI (Fig. 14), hip muscle CCI significantly decreased after EMS training, whereas ankle muscle CCI remained unchanged. This reduction in hip CCI suggests more efficient coordination between the quadriceps and hamstrings, supporting greater reliance on hip-mediated control during the task.

These findings suggest that EMS training led to a hip-centered and more flexible coordination pattern during the dynamic balance task. The reduction in hip muscle CCI, together with the maintenance of pelvic motion, indicates decreased joint stiffness and greater reliance on controlled hip motion for board stabilization. In contrast, the reduction in pelvic sway observed in the control group suggests a tendency toward a stiffness-based strategy, possibly involving increased rigidity at the hip joint to constrain motion. Additionally, the reduced sway near the shoulders in the EMS group suggests the use of hips as the primary point of control while the feet and shoulders act as stabilizing anchors. Thus, while overall task performance was similar, the coordination patterns differed substantially between groups.

### Integration of ST and BB findings: relationship between sensory reweighting and control strategy

EMS-based balance training was associated with a shift in sensory weighting toward greater visual reliance during ST task. When integrating findings across both ST and BB tasks, the results indicate a transition from ankle-dominant, stiffness-based control toward a more flexible, hip-centered multi-joint coordination strategy. These sensory and strategic adaptations appear to be functionally related. EMS-assisted ankle movement may have reduced the need for voluntary ankle control, thereby shifting control toward proximal joints, particularly the hip. Such a strategy likely promoted stabilization of the trunk, especially around the shoulder segment. Reduced sway of the upper body would in turn decrease oscillations of the head and visual field, thereby facilitating more effective use of visual information for postural control. Notably, while the BB task and training sessions targeted control in the AP direction, the increased COP sway and visual weighting were observed in the ML direction. This directional difference may be related to the ${45}^{\circ }$ foot angle used in the ST task; in this posture, the control strategy in the AP direction is primarily ankle-dominant, whereas the ML direction involves a combination of ankle and hip strategies [50, 51]. As discussed in Section "Effects of EMS-based balance training on the BB task", EMS-based training during the BB task suggested greater involvement of hip control, which may be more readily manifested in the ML plane given the ${45}^{\circ }$ stance configuration of the ST task, thereby accounting for the observed changes in postural sway and sensory weighting.

We assume that this relationship arises from the unique characteristic of EMS in producing involuntary muscle contractions. EMS applied to agonist muscles is known to facilitate reciprocal inhibition of antagonist muscles and has been utilized in spasticity rehabilitation and flexibility training contexts [52, 53]. Moreover, we previously reported that EMS during balance board standing may enhance the efficiency of agonist–antagonist switching [18]. It is therefore plausible that externally assisted ankle activation accelerated local muscle responses, requiring compensatory coordination of proximal joints. Such adaptations may have contributed to the observed changes in multi-joint control patterns and their associated sensory reweighting dynamics.

### Effects of EMS-based balance training on postural control improvement and clinical implications

The present findings demonstrate that EMS-based balance training transiently modulates sensory weighting and postural control strategies. However, whether these adaptations directly translate into overall postural improvement remains unclear. By examining both beneficial and potentially adverse aspects of these short-term changes, the results provide insights for developing individualized, sensory-augmented training approaches.

EMS training increased visual reliance during the ST task. Sensory weighting differs substantially across individuals [54, 55], and the optimal sensory contribution strategy depends on the individual’s condition and task demands. Even among older adults, sensory reweighting capacity is heterogeneous, reflecting differences in neuroplasticity and comorbidities [10]. In sports settings, sensory orientation often reflects discipline-specific demands [56]. From this perspective, EMS-induced reweighting may provide a means to transiently rebalance sensory contributions, particularly in individuals with insufficient visual reliance or excessive dependence on other modalities. In clinical populations with partial sensory deficits, such as peripheral neuropathy or vestibular dysfunction, promoting greater use of intact visual input may support early adaptation. Nevertheless, increased visual dependence may also present drawbacks. Because visual processing involves longer latencies [2], greater reliance on vision may delay corrective responses to perturbations. Notably, the increased COP sway observed under the ECRP condition suggests that stability may be compromised in visually constrained or challenging contexts, particularly in individuals who are already visually dependent.

EMS training also facilitated a shift from ankle-dominant, stiffness-based control toward a hip-centered, multi-joint coordination strategy characterized by greater movement complexity. Low-complexity, co-contracted strategies are commonly observed under high task difficulty and in older or balance-impaired individuals [34, 57, 58], and are considered less efficient [59, 60]. In contrast, higher movement complexity is associated with expert performance and more automatic control processes [61, 62]. Thus, EMS may promote more flexible and redundant postural coordination. However, excessive reliance on hip-dominant strategies could reduce rapid ankle-mediated corrections, potentially lowering responsiveness to sudden perturbations.

Overall, the impact of EMS training on postural improvement likely depends on individual sensory-motor profiles. While the intervention appears capable of inducing flexible strategy shifts, its benefits are not universal. These implications should be interpreted cautiously, as the findings were obtained in young healthy adults and reflect short-term adaptations. Further research is needed to determine their long-term and clinical relevance.

### Limitations

This study has several limitations. First, the present findings do not demonstrate a general improvement in postural balance. The observed changes should be interpreted as short-term modifications in sensory weighting and coordination patterns rather than definitive evidence of enhanced stability. Whether these adaptations result in functional improvement remains to be determined. Moreover, the participant characteristics were limited, and further studies are required to determine whether the effects observed here can be generalized to other populations. Second, it remains unclear whether participants indeed controlled the balance board primarily through hip strategies during the EMS-based training session because EMG could not be recorded during EMS presentation. This constraint leaves open the possibility that the observed changes reflect a short-term after-effect, with participants needing to readjust to the condition without EMS, rather than an adaptive training effect. In addition, because muscle force does not cease instantaneously after the termination of activation due to muscle relaxation dynamics [63], the assisted ankle movements may have occasionally continued after EMS cessation, potentially requiring additional voluntary corrections. Furthermore, the EMS parameters used in this study, particularly the high frequency of 200 Hz, present a potential limitation regarding muscle fatigue. Future rehabilitation applications would require strategies to mitigate muscle fatigue and may need optimization of stimulation parameters. Next, the degree of improvement in balance board sway did not differ between groups, possibly due to the relatively high baseline balance ability of the participants. It remains necessary to evaluate whether practice leads to measurable improvements in balance performance among individuals with lower balance capacity. Finally, the absolute values of sensory weighting remain unknown. Although neuroimaging approaches such as EEG or fNIRS might provide additional insight, measuring brain activity during balance tasks remains technically challenging. Nevertheless, collecting data on sensory dependence in large populations may help to capture relative sensory characteristics, potentially leading to the implementation of a consistent program that spans from the assessment of individual sensory dependence to the practical application of balance training.

## Conclusion

In this study, we demonstrated that EMS-based balance board training can induce modifications in sensory reweighting dynamics and postural control strategies. Specifically, providing EMS to the ankle muscles, to assist in maintaining the board horizontally, induced post-training adaptations characterized by inducing visual reweighting and a transition to a multi-joint strategy involving more flexible use of the hip joint. Furthermore, the involuntary assistance uniquely provided by EMS may have been related to changes in postural control strategies that, in turn, modulated visual reweighting dynamics. These findings represent the first evidence that incorporating EMS into unstable balance tasks can induce specific adaptations in both sensory reweighting dynamics and postural control strategies. Such EMS-based approaches will provide a foundational knowledge for training methods that shift postural control toward greater visual reliance. For establishing a novel balance training that selects optimal feedback methods according to individual sensory characteristics, future research should continue to explore augmented feedback techniques that induce other forms of sensory reweighting.

## Additional file

Supplementary Material 1. Statistical results for all postural control outcome measures.

## Data Availability

All data generated or analyzed during this study are included in this published article and its supplementary information files.

## Abbreviations

AP: Anteroposterior
BB: Balance board
CCI: Co-contraction index
CCR: Cross-correlation coefficient
COP: Center of pressure
EC: Eyes closed
ECRP: Eyes closed on foam rubber pad
EO: Eyes open
EORP: Eyes open on foam rubber pad
EMG: Electromyography
EMS: Electrical muscle stimulation
GA: Gastrocnemius HA: Hamstrings
ML: Mediolateral
PSD: Power spectral density
RF: Rectus femoris
RMS: Root mean square
SampEn: Sample entropy
SO: Soleus
ST: Static standing
TA: Tibialis anterior
